# Discovery of Novel Potential Prognostic Markers and Targeted Therapy to Overcome Chemotherapy Resistance in an Advanced-Stage Wilms Tumor

**DOI:** 10.3390/cancers16081567

**Published:** 2024-04-19

**Authors:** Pongsakorn Choochuen, Natakorn Nokchan, Natthapon Khongcharoen, Wison Laochareonsuk, Komwit Surachat, Thirachit Chotsampancharoen, Thanit Sila, Surasak Sangkhathat Consortium

**Affiliations:** 1 Department of Biomedical Sciences and Biomedical Engineering, Faculty of Medicine, Prince of Songkla University, Songkhla 90110, Thailand; pongsakorn.c@psu.ac.th (P.C.); natakorn.n@psu.ac.th (N.N.); 6510330010@psu.ac.th (N.K.); wison.l@psu.ac.th (W.L.); komwit.s@psu.ac.th (K.S.); 2 Translational Medicine Research Center, Faculty of Medicine, Prince of Songkla University, Songkhla 90110, Thailand; 3 Department of Pediatrics, Faculty of Medicine, Prince of Songkla University, Songkhla 90110, Thailand; cteerach@medicine.psu.ac.th; 4 Department of Pathology, Facualty of Medicine, Prince of Songkla University, Songkhla 90110, Thailand; thanit.s@psu.ac.th; 5 Department of Surgery, Faculty of Medicine, Prince of Songkla University, Songkhla 90110, Thailand

**Keywords:** Wilms tumor, chemotherapy resistance, biomarkers, somatic mutation, targeted therapy, cancer driver mutation

## Abstract

**Simple Summary:**

Resistance to chemotherapeutic drugs poses a significant challenge in the treatment of Wilms tumor (WT), contributing to cancer recurrence and compromising patient survival. This study aims to elucidate potential mutation markers and drug targets associated with chemotherapy resistance in advanced-stage WT. Through comprehensive exome sequencing analysis, we identified variants in four genes—*ALPK2*, *C16orf96*, *PRKDC*, and *SVIL*—that exhibit strong correlations with chemotherapy resistance. These variants are also associated with reduced disease-free survival in advanced-stage WT and hold promise as potential prognostic markers. Furthermore, by characterizing druggable mutations, we propose novel therapeutic strategies involving platinum-based agents, PARP inhibitors, and antibiotic/antineoplastic agents. These findings pave the way for innovative alternative therapies to combat chemotherapy resistance in advanced-stage Wilms tumors.

**Abstract:**

Wilms tumor (WT), the most prevalent type of renal cancer in children, exhibits overall survival rates exceeding 90%. However, chemotherapy resistance, which occurs in approximately 10% of WT cases, is a major challenge for the treatment of WT, particularly for advanced-stage patients. In this study, we aimed to discover potential mutation markers and drug targets associated with chemotherapy resistance in advanced-stage WT. We performed exome sequencing to detect somatic mutations and molecular targets in 43 WT samples, comprising 26 advanced-stage WTs, of which 7 cases were chemotherapy-resistant. Our analysis revealed four genes (*ALPK2*, *C16orf96*, *PRKDC*, and *SVIL*) that correlated with chemotherapy resistance and reduced disease-free survival in advanced-stage WT. Additionally, we identified driver mutations in 55 genes within the chemotherapy-resistant group, including 14 druggable cancer driver genes. Based on the mutation profiles of the resistant WT samples, we propose potential therapeutic strategies involving platinum-based agents, PARP inhibitors, and antibiotic/antineoplastic agents. Our findings provide insights into the genetic landscape of WT and offer potential avenues for targeted treatment, particularly for patients with chemotherapy resistance.

## 1. Introduction

Wilms tumor (WT), also known as nephroblastoma, is the most prevalent renal tumor in children, accounting for more than 90% of cases. It is also the second most frequent extracranial tumor after neuroblastoma. A global study found that the age-adjusted incidence rate of renal tumors in children was 8.3 per million and that this rate showed an upward trend, particularly in Southeast Asia [1]. In the United States, approximately 600 new cases are diagnosed annually, and the incidence appears to be relatively stable [2]. The treatment of WT generally has a favorable prognosis, with an overall survival rate of over 90%. However, 10% of WT cases are resistant to chemotherapy and have poor outcomes [3]. Moreover, disease recurrence is another key factor that influences patient survival. The survival rate for recurrent WT after treatment is only 25%, even with intensive chemotherapy [4]. WT at advanced stages (COG stage III–V) has a higher risk of recurrence than WT at lower stages (COG stage < II). The 4-year event free survival for COG stage III and stage IV WT was 80.9% and 41.7%, respectively, while it was 86% for COG stage II WT [3]. Current research on WT focuses on identifying molecular biomarkers and investigates molecular-targeted therapies as possible strategies to overcome chemotherapy resistance [5].

WT exhibits molecular pathogenesis involving the activation of Wnt signaling pathways. This is supported by the detection of somatic variants in *CTNNB1* (10–15%), coupled with background germline variants in *WT1*. These two genes are normally involved in the renal development process [6,7]. Additionally, somatic variant in the tumor suppressor gene *TP53* can be identified in 47.5% of WT patients with diffuse anaplastic histology. Other somatic variants found in WT include *DROSHA*, *AMER1* (*WTX*), and *DGCR8*, which occur in around 5–10% of WT cases [8]. Furthermore, somatic variant in genes associated with the microRNA biogenesis process (*DGCR8*, *DICER1*, *XPO5*, and *TARBP2*) were found in up to 12% of WTs [9,10]. Interestingly, *AMER1* variants have a high frequency (10.1%), but it does not appear to have any discernible clinical impact. However, *WT1* could be a potential target in anti-angiogenesis therapy, such as bevacizumab and AZD2171 [11]. Although targeted therapy for *TP53* has not been specifically mentioned in WT, drugs targeting this gene with FDA approval and in clinical trials are commonly available [12]. Unfortunately, few drugs have been developed specifically for childhood tumors due to a small market for a rare childhood disease. Current research on WT treatment mainly focused on repurposing pediatric tumor-specific targeted therapy and immunotherapy. These two types of drugs were believed to become the two major adjuvant treatment for postoperative WT [11]. While most of the other variants are associated with clinical outcomes and treatment [13], none of these variants have been used as markers for chemotherapy resistance or molecular targets for therapy. The identification of somatic variants in chemotherapy-resistant WT implies the possibility of finding mutation markers for predicting resistance in WT cases and discovering new molecular biomarkers and therapeutic targets to overcome chemotherapy resistance in WT [14,15]. This study aimed to investigate the somatic mutational landscape in WT using exome sequencing, identify potential mutation markers for predicting chemotherapy resistance events in advanced-stage WT, and suggest the targeted chemotherapy based on the profiles of driver mutations that might serve as novel therapeutic strategies against chemotherapy resistance.

## 2. Materials and Methods

### 2.1. Biological Samples and Sequencing Library Preparation

**Figure 1:**
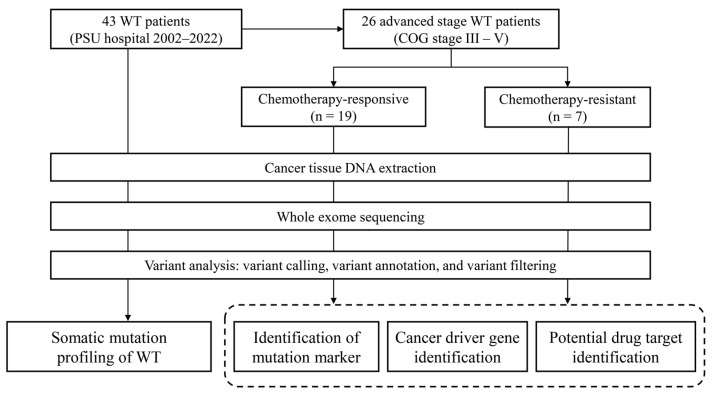
Overview of the workflow. Cancer tissues were obtained from 43 cases of WT. Tissue-derived DNA was extracted and subsequently underwent whole exome sequencing (WES). Variants detected through WES data analysis were filtered. The resultant variants were employed for downstream analysis.

We obtained 43 fresh frozen tissue samples of WT from the biological repository of the Translational Medicine Research Center, Faculty of Medicine, Prince of Songkla University (Songkhla, Thailand). Unfortunately, their corresponding normal adjacent tissues or blood samples were unavailable among our cases. These samples originated from the surgical specimens of 43 WT patients who were younger than 15 years old and had had been diagnosed with primary renal tumors. These patients received nephrectomy at Songklanagarind Hospital between 10 January 2003 and 26 December 2020. DNA extraction was performed using DNeasy Blood and Tissue Kit (Cat. No.: 69504; Qiagen Inc., Redwood City, CA, USA). Subsequently, the quantity and quality of the extracted DNA were assessed using Nanodrop (Thermo Fisher Scientific, Inc., Waltham, MA, USA) and TapeStation (Agilent Technologies, Inc., Santa Clara, CA, USA). The clinical information of all the patients was retrieved from the electronic medical records (EMR) of the hospital. The EMR review process was conducted from 1 April 2022 to 31 May 2022. All patients and their legal guardians provided written informed consent before recruitment. This study was approved by the Ethical Committee of Faculty of Medicine, Prince of Songkla University (REC 64-195-10-1) (Figure 1).

### 2.2. Whole Exome Sequencing

Our study employed whole exome sequencing as we primarily focused on the comprehensive analysis of the protein-affecting somatic variant. Whole exome sequencing was conducted using the library preparation system of Agilent SureSelect XT Human All Exon v6 (Agilent Technologies, Inc.) according to the manufacturer’s specifications. The library was quantified using a Qubit dsDNA High Sense Assay Kit (Invitrogen, Carlsbad, CA, USA), and size measurements were performed using the Agilent D1000 ScreenTape assay. Sequencing was performed using an Illumina NovaSeq-6000 platform (Illumina, San Diego, CA, USA) with paired-end reads of 150 bp. The average targeted coverage depth achieved was 200×.

The paired-end sequence files were assessed for quality using FastQC (version 0.11.9) and trimmed using Trimmomatic (version 0.39) [16]. The optimally prepared FASTQ files were aligned with the human reference genome (version GRCh38.13) using the BWA program (version 0.7.17) [17]. The resulting Sequence Alignment Map (SAM) files were converted to a Binary Alignment Map (BAM) format and sorted using SAMtools (version 1.17) [18]. Subsequently, the sorted BAM files were regrouped, and identical sequences were marked using Picard (version 3.0.0). To adjust the base quality score, unduplicated BAM files were processed using the Genomic analysis toolkit (GATK, version 4.4.0) base quality score recalibration [19]. The GATK tool was subsequently employed for somatic variant discovery, adhering to standard practices in genomic variant calling for both research and clinical contexts [20]. Variant calling was performed using Mutect2 in tumor-only mode. A public Panel of Normals (PON) was downloaded from the public repository of GATK via https://storage.googleapis.com/gatk-best-practices/somatic-hg38/1000g_pon.hg38.vcf.gz, accessed on 9 November 2022. We filtered the variants generated by Mutect2 using GATK4 GetPileupSummaries, CalculateContamination, and FilterMutectCalls with a default value of argument. The resultant variants were then annotated using Funcotator. The annotated mutational data were stored in MAF files. The files were subsequently summarized and visualized using maftools package in R [21].

### 2.3. Identification of Mutation Marker for Chemotherapy-Resistant WT

Patients with advanced-stage WT was divided into two groups according to their responses to standard postoperative chemotherapy following nephrectomy, as per the Thai Pediatric Oncology Group (ThaiPOG) protocol [22]. Chemotherapy resistance was defined as either no change or an increase in tumor size after chemotherapy or recurrence during chemotherapy. Both primary and metastatic tumors were considered for the chemotherapy resistance assessment. In our study, 19 patients with advanced-stage WT responded to chemotherapy, while 7 were resistant. The proportion of affected patients with somatic mutations in each gene across two groups was compared using Fisher’s exact test. A *p*-value < 0.05 was considered to be statistically significant. We also performed association analysis between the somatic mutation profile and other clinical parameters, including histology, syndromic features, and lesion laterality, using the same method.

### 2.4. Identification of Cancer Driver Mutation and Their Potential Targeted Therapy

Driver mutation is defined as the mutation that confers a growth advantage to cancer cells, enabling cancer initiation and progression [23,24]. We prioritized the annotated somatic mutations from the chemotherapy-resistant group to identify potential driver mutations using two web-based machine learning tools, BoostDM and OncodriveMUT, accessible from the Cancer Genome Interpreter (CGI, https://www.cancergenomeinterpreter.org, accessed on 16 November 2022) [25]. These tools have accuracy above 0.8 in detecting driver variants. In particular, BoostDM outperformed the other tools [26]. The CGI database also provided the potential target therapy effective against the identified driver mutation. Additionally, we searched the chemotherapy data, clinical trial studies, and level of evidence from MSK’s Precision Oncology Knowledge Base (OncoKB, https://www.oncokb.org, accessed on 16 November 2022) and the Clinical Interpretation of Variants in Cancer (CIVIC, https://civicdb.org, accessed on 16 November 2022).

### 2.5. Statistical Analysis

Statistical analyses were conducted using RStudio, based on the statistical language R version 4.3.0. We utilized Fisher’s exact test to assess the association between genes with somatic mutations and patients’ clinical phenotypes. Specifically, we compared the proportion of patients with somatic mutations and wild-type genes to the proportion of patients exhibiting the phenotype of interest. Survival analysis was performed using the Kaplan–Meier survival probability for survival function and the log-rank test for survival comparison. The Mantel–Haenszel method was used to estimate the hazard ratio for each identified marker. The level of statistical significance was set at *p*-value < 0.05.

## 3. Results

### 3.1. Clinical Characteristics of the Patients

**Figure 2:**
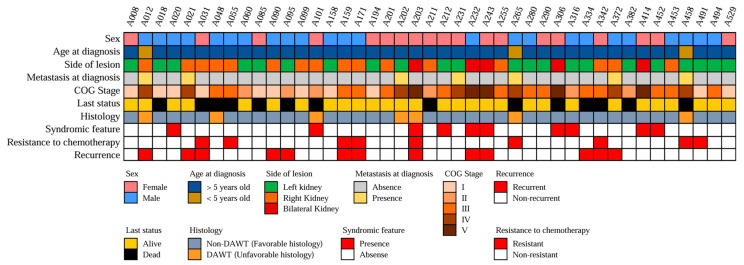
Clinicopathological feature of WT patients Heatmap representing the clinicopathological attributes of 43 patients with WT. Various clinicopathological traits are denoted by distinct color codes. The classification of patients as having a “syndromic feature” is based on the presence of at least one of the following anomalies: genitourinary tract anomalies, aniridia, albinism, or congenital spine anomalies.

The analysis of 43 pediatric WT tissue samples was performed in this study. Figure 2 shows the clinical features of all the patients. The diagnosis was made at a median age of 20.2 months (IQR: 10.9–39.5 months). The primary tumor was located on the left side in 24 cases (48.9%), on the right side in 20 cases (40.8%), and bilaterally in five cases (10.2%). The COG staging system classified 11 WT cases (25.6%) as stage I, six (14.0%) as stage II, 14 (32.6%) as stage III, seven (16.3%) as stage IV, and five (11.6%) as stage V. Thus, advanced-stage disease (stage III–V) accounted for 60.47% (*n* = 26) of all cases. The ThaiPOG protocol recommended a combination of vincristine and actinomycin D as the postoperative chemotherapy regimen for localized WT, and Doxorubicin, cyclophosphamide, and etoposide may be added for patients at stages III–IV. This study revealed that nine WT cases (20.93%) were resistant to the aforementioned chemotherapy regimen, of which seven cases (16.28%) had advanced-stage disease (the chemotherapy regimen for these patients can be found in the Supplementary Data). Furthermore, this study detected recurrence disease in 13 cases (30.23%), despite standard surgery and chemotherapy. Additionally, ten patients displayed clinical phenotypes that could be linked to cancer syndromes, such as genitourinary tract anomalies, aniridia, albinism, and congenital spine anomalies.

### 3.2. Somatic Mutation Profiling of WT and Clinical Relevance

**Figure 3:**
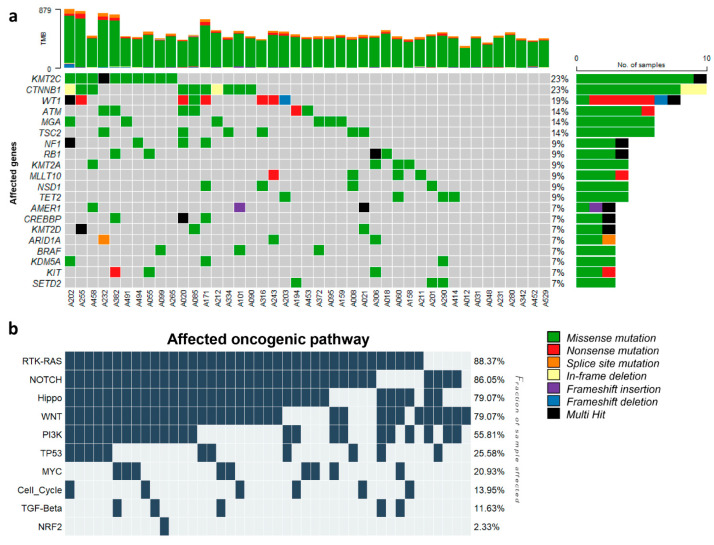
Somatic mutation profile and affected biological pathway in WT. (**a**) Oncoplot depicting somatic mutations in commonly known pediatric cancer-related genes among 43 patients with WT. The top histogram displays the tumor mutation burden (TMB), which is the number of somatic mutations per sample. The right bar chart indicates the percentage of samples with mutations in each gene. (**b**) Oncoplot illustrating the 10 most frequently affected pathways. The pathway names are indicated on the left, and the proportions of patients with mutations in each pathway are shown on the right.

The bioinformatics analysis of the exome sequences of 43 WT samples identified 15,492 single-nucleotide variants (SNVs) and 295 insertions or deletions (indels). These variants were exclusively exonic non-synonymous variants with a high or moderate impact and a coverage of at least 20×. The analysis detected mutations in several cancer-related genes previously reported to be implicated in pediatric cancer, such as CTNNB1, WT1, AMER1, and NF1. These genes were also listed among the 15 most frequently mutated genes in this analysis, which include KMT2C, CTNNB1, WT1, ATM, MGA, TSC2, NF1, RB1, KMT2A, MLLT10, NSD1, TET2, AMER1, CREBBP, and KMT2D (Figure 3a). Among them, CTNNB1, WT1, and AMER1 have been identified in large cohorts of WT cases. Additionally, NF1 and CREBBP were previously reported in a single WT case. Although the remaining ten genes have not been previously reported to be mutated in WT, they have been associated with other pediatric malignancies. TSC2 and RB1 genes have been found to be mutated in acute T-cell lymphoid leukemia and osteosarcoma. Mutations in ATM, MGA, KMT2A, MLLT10, NSD1, and TET2 have been commonly identified in several hematological neoplasms, including both acute lymphoid and myeloid leukemia [27,28]. KMT2C and KMT2D were frequently mutated in multiple types of cancer [29,30].

Furthermore, we analyzed the KEGG pathways of 43 patients with WT and identified the top 10 biological pathways affected by genetic alterations (Figure 3b). Six pathways were affected by at least one mutated gene in all 43 WTs. All were signaling pathways, including the RTK-RAS, WNT, NOTCH, Hippo, PI3K, and MYC signaling pathways. The activation of the Wnt/β-catenin pathway is commonly associated with WTs [31]. The aberrant activation of the PI3K-Akt signaling pathway, which plays a crucial role in cell proliferation, differentiation, and growth, [32], has been identified in different types of leukemia and solid tumors, including WT [33,34]. The mutation of genes in the MYC signaling pathway, namely MYCN and MAX, has been found to be strongly associated with an increased risk of WT relapse [35]. However, the remaining pathways were cancer-related signaling pathways that are commonly found in several types of human cancer [36,37].

We conducted a study to investigate the association between somatic variants and clinical parameters in our cohort. We used Fisher’s exact test to evaluate the association and found significant associations (Supplementary Table S1). Specifically, we found that syndromic feature was associated with variant detection in 38 genes, and tumor laterality was linked to 10 genes. We also identified seven genes, including *PRSS2*, *PRSS1*, *AKR7A3*, *ARID1A*, *METTL14*, *BRD8*, and *CHD8*, that were associated with both syndromic feature and the bilaterality of tumors.

**Figure 4:**
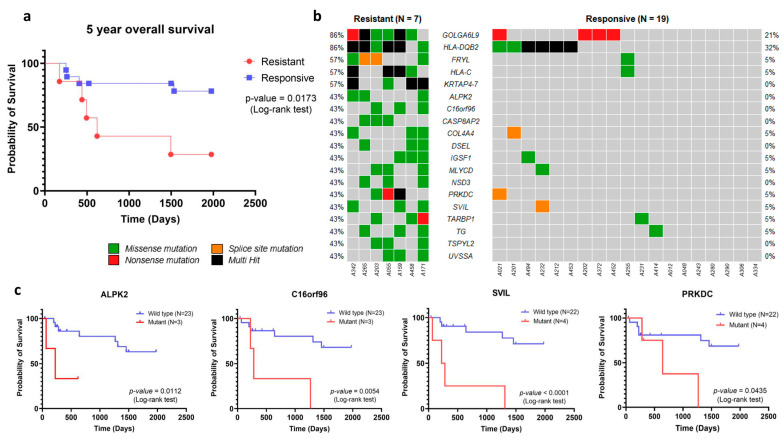
Association between somatic mutation and chemotherapy resistance in WT. (**a**) Comparing the five-year overall survival of advanced WT patients with different chemotherapy responses. (**b**) An oncoplot displaying the somatic mutations of a gene that was significantly associated with chemotherapy resistance in each patient from both resistant and responsive groups. (**c**) The Kaplan–Meier method estimated the disease-free survival of patients with mutations in four genes (*ALPK2*, *C16orf96*, *SVIL*, and *PRKDC*), which were associated with shorter survival compared to wild-type patients.

Furthermore, we found that 23 genes were associated with unfavorable histology, among which *MMP17*, *OR6C70*, and *MPDZ* variants were only present in unfavorable histology with the lowest *p*-value. In addition, we focused on the therapeutic response of WT in our cohort. We found that from all advanced-stage WT (*n* = 26), 7 cases were resistant to postoperative chemotherapy. The median time of resistance was 190 days (ranging from 72 to 320 days) after surgery. Resistance to chemotherapeutic drug is a significant problem in cancer therapy, as it causes most cases of cancer recurrence and reduces the chances of survival for patients [38]. The 5-year overall survival of advanced-stage WT patients with chemotherapy resistance (*n* = 7) indicated that the median time of survival in this group was 619 days, with a corresponding hazard ratio (HR) calculated using the Mantel–Haenszel method at 6.75 (95% confidence interval (CI): 1.40–32.53). These results demonstrated significantly poorer survival in the chemotherapy resistance group compared to that of the chemotherapy-responsive patients (*n* = 19) (Figure 4a, *p*-value = 0.0173). The poorer 5-year overall survival of advanced-stage WT patients with chemotherapy resistance emphasized its impact on patient survival probability. To identify genetic markers for chemotherapy resistance, an association analysis using Fisher’s exact test was carried out to determine the relationship between genetic variants and chemotherapy resistance. Significant associations were found for variants in 19 genes that were associated with chemotherapy resistance (*p*-value < 0.05, Figure 4b). When we used more stringent criteria at a *p*-value < 0.01, we could identify four genes, including *KRTAP4-7*, *GOLGA6L9*, *FRYL*, and *HLA-C*, that were associated with chemotherapy resistance (Table 1).

In the analysis of disease-free survival, we identified variants in four genes, namely *ALPK2*, *C16orf96*, *PRKDC*, and *SVIL*, that were significantly associated with lower disease-free survival (Figure 4c). We estimated the hazard ratio of these genes using the Mantel–Haenszel method. The results showed that *ALPK2* (HR, 60.04 [95% CI: 2.534–1423]), *C16orf96* (HR, 31.20 [95% CI: 2.765–351.9]), *PRKDC* (HR, 8.438 [95% CI: 1.064–66.94]), and *SVIL* (HR, 63.82 [95% CI: 6.679–609.8]) were found to independently predict survival outcomes. Notably, these four genes might serve as putative candidate markers for predicting chemotherapy resistance among advanced-stage WT patients.

### 3.3. Cancer Driver Gene and Potential Drug Target for Chemotherapy-Resistant WT

**Figure 5:**
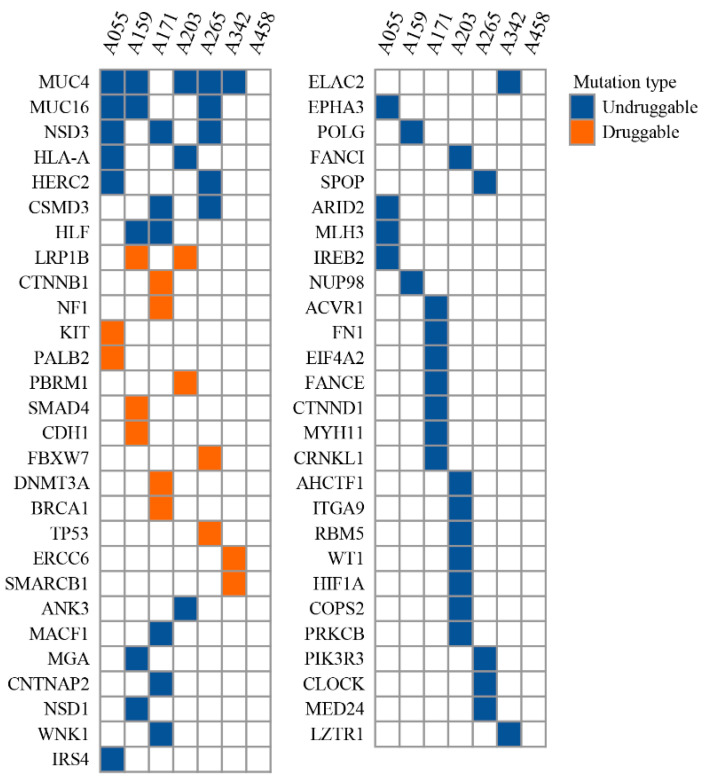
Cancer driver genes identified in chemotherapy-resistant WT. The distribution of driver mutations identified in advanced-stage WT patients with chemotherapy resistance. The mutations were classified as druggable or undruggable based on the availability of targeted therapies. The druggable mutations were marked with orange color, while the undruggable mutations were marked with blue color.

The analysis could identify driver mutations in 55 genes (Supplementary Figure S1). Among those, eight driver genes were the most commonly recurrent driver, including *MUC4, MUC16*, *NSD3*, *HLA-A*, *HERC2*, *CSMD3*, *HLF*, and *LRP1B*, which could be identified in at least two WT cases each (Figure 5). Driver mutations in *MUC4* and *MUC16* were the two most commonly identified. These two genes belong to the membrane-bound mucins family. Studies have reported a regulatory relationship between these two mucins and the canonical Wnt/β-catenin signaling pathway, which is a common pathway found to be activated in WT [39,40,41]. Although the association between these two mucins and β-catenin has been reported in many cancers, including pancreatic, lung, colorectal, and ovarian cancer, this relationship in WT is less known [42]. Moreover, the identification of Wnt/β-catenin as a pathway associated with WT and the mutation of *MUC4* and *MUC16* among WT cases might hint at a relationship between β-catenin and these two mucins.

To discover the potential drug target of our identified driver mutation, the annotated drivers were examined for their potential association with molecular-targeting drugs, as determined by the Cancer Genome Interpreter. The analysis could identify druggable mutation on 14 cancer driver genes (Supplementary Table S2). Notably, platinum-based chemotherapeutic agents, namely Cisplatin and Carboplatin, have favorable responses in cancer with the presence of three driver mutations: *PALB2* (p.M723X), *BRCA1* (p.Q262H), and *ERCC6* (p.M867V). In our cohort of resistant patients, 43% (3 out of 7) had mutations in these genes. Although the effect of platinum-based agents on WT with mutations in these genes has not been evaluated, previous clinical trials have investigated the responses of mentioned agents in other cancers, such as breast, ovarian, and pancreatic cancer, that harbor the same driver mutations as our WT cases [43,44,45,46]. Therefore, these agents may be potential candidates for overcoming chemotherapy resistance in WT. In addition to platinum-based agents, PARP inhibitors are effective treatments for cancer patients with mutations in *PALB2* (p.M723X) or *BRCA1* (p.Q262H) genes. Several studies have demonstrated the clinical benefits of PARP inhibitors for these patients, and the US FDA has approved their use for breast [47,48], ovarian [49], and prostate cancer [50,51,52] patients with *PALB2* or *BRCA1* mutations.

Antibiotic/antineoplastic agents, such as Daunorubicin, Doxorubicin, and Mytomycin C, are promising chemotherapeutic agents that could be the preferred therapy for chemotherapy-resistant WT. These agents interfere with DNA synthesis as their mechanism of action. Clinical trials have confirmed the effectiveness of Daunorubicin in treating acute myeloid leukemia with the *DNMT3A* (p.V687F) mutation, and professional guidelines have endorsed its use [53]. Early trial studies have also demonstrated the responsiveness of Mytomycin C in pancreatic cancer with *PALB2* (p.M723X) [54]. Furthermore, the CGI database has documented the efficacy of Doxorubicin against breast adenocarcinoma with *TP53* (p.R273C) in the CGI database. However, liposomal Doxorubicin was found to be ineffective in ovarian cancer with *LRP1B* (p.S1148P and p.W3333L) [55]. These findings indicate the potential of antibiotic/antineoplastic agents in treating WT cancer with the driver mutations that we also detected in 43% (3 out of 7) of our patients. Another druggable driver mutations that were identified in chemotherapy-resistant WT are *PBRM1* (p.G989C) and *CDH1* (p.D433G), which were responsive to EZH2 inhibitors and an AR inhibitor (Bicalutamide), respectively [56,57]. Each variant was detected in a different patient out of the seven in our cohort. However, the effectiveness of these drugs has only been reported in pre-clinical studies.

The findings from our investigation of variant markers and alternative drugs for chemotherapy-resistant WT offer a novel perspective on the treatment of this condition. However, it is important to note that these results were derived from a small patient cohort. To enhance the validity of our study, further validation in a larger clinical cohort would be beneficial.

## 4. Discussion

Studies on somatic mutations in various cancers have led to the identification of molecular targets that might suggest novel therapies for the diseases, either through the development of new drugs or by repurposing existing ones. Molecular targeted therapy is an essential part of modern cancer treatment, but the options for WT are limited in terms of molecular drugs. This is due to the low incidence and distinct pathogenesis of these tumors. The analysis detected many genetic variants and mutations in genes related to cancer, including *CTNNB1* and *WT1*, which have been previously reported to be associated with WT [6,7]. Consistent with the analysis result, *CTNNB1* and *WT1* were found in 23% and 19% of our cohort, respectively. The genetic alteration profile revealed 15 genes that were most frequently mutated in our WT patients. Among these genes, five genes have previously been identified in WT tumor. The other ten genes have not been reported to be mutated in WT before, but they have been associated with various pediatric cancers [27,28,29,30]. The analysis also showed the top 10 biological pathways that were affected by the genetic alterations, most of which were signaling pathways that regulate cell growth and survival, corroborating the pathogenesis process of this malignancy.

Previous studies have reported that about 10% of WT patients exhibit resistance to standard chemotherapy, leading to poor prognosis [3]. Furthermore, disease recurrence is another major factor affecting patient survival. It has been shown that the survival rate for recurrent WT was only 25% despite aggressive chemotherapy [4]. These findings indicate the importance of chemotherapy resistance and disease recurrence as predictors of poor disease outcome. In our study, we found that advanced-stage WT patients with chemotherapy resistance (*n* = 7) had significantly lower 5-year overall survival than advanced-stage WT who responded to chemotherapy (*n* = 19). The prediction of drug resistance in patients before chemotherapy administration could facilitate the selection of alternative chemotherapy regimens and improve patient prognosis. Our association analysis identified variants in 19 genes that were associated with chemotherapy resistance in advanced-stage WT. Among them, variants in four genes, *ALPK2*, *C16orf96*, *PRKDC*, and *SVIL*, were significantly associated with lower disease-free survival. However, additional validation studies are necessary to recruit more clinical samples and assess their predictive power. In this validation study, we recommend using a simple sequencing method, such as capillary DNA sequencing, to enhance the clinical utility of the marker.

To find a therapeutic agent that could overcome chemotherapy resistance in WT, we first identified driver mutations in advanced-stage WT with chemotherapy resistance. We found recurrent driver mutations in 55 genes, of which 14 were cancer driver genes with available drugs. Notably, 43% (3 out of 7) of the resistant WT cases had at least one driver mutation in *PALB2* (p.M723X), *BRCA1* (p.Q262H), or *ERCC6* (p.M867V). These mutations have been shown to be predictive of sensitivity to platinum-based chemotherapeutic agents in several clinical trials. Furthermore, cancers with *PALB2* (p.M723X) and *BRCA1* (p.Q262H) mutations can also benefit from PARP inhibitors, which are recommended in professional guidelines for treating breast, ovarian, and prostate cancer [47,48,49,50,51,52].

Besides the aforementioned drugs, antibiotic/antineoplastic agents, such as Daunorubicin, Doxorubicin, and Mytomycin C, can also target cancers with *DNMT3A* (V687F), *PALB2* (M723X), and *TP53* (R273C) mutations [53,54,55]. These mutations were also present in 43% (3 out of 7) of the resistant WT cases. Other druggable driver mutations that we detected in resistant WT cases were *PBRM1* (p.Gly989Cys) and *CDH1* (p.Asp433Gly), which were responsive to EZH2 inhibitors and AR inhibitor (Bicalutamide), respectively [56,57]. Although the efficacy of these chemotherapeutic agents on WT with the same mutations as ours has not been assessed, previous clinical trials have demonstrated their activity in other cancers with the same driver mutations. Moreover, this lack of assessment also affects our understanding of drug safety, as there is limited information available for WT patients. Consequently, further research is needed to evaluate the therapeutic potential of targeting the driver mutations that we identified in our advanced-stage WT with chemotherapy resistance.

## 5. Conclusions

In summary, we performed exome sequencing to discover somatic variants in 43 WT cases. Our findings reveal the possible mutation markers for chemotherapy resistance in advanced-stage WT and suggest the potential therapeutic options that could be effective for this group of patients.

## Figures and Tables

**Table 1:** The mutation rate in the genes associated with chemotherapy resistance.

Gene	Chemotherapy Response *		*p*-Value
	Resistant (N:7)	Responsive (N:19)	
*KRTAP4-7*	4/7 (51.14%)	0/19 (0.00%)	0.002
*GOLGA6L9*	6/7 (85.71%)	4/19 (21.05%)	0.005
*FRYL*	4/7 (51.14%)	1/19 (0.05%)	0.010
*HLA-C*	4/7 (51.14%)	1/19 (0.05%)	0.010
*ALPK2*	3/7 (42.86%)	0/19 (0.00%)	0.013
*C16orf96*	3/7 (42.86%)	0/19 (0.00%)	0.013
*CASP8AP2*	3/7 (42.86%)	0/19 (0.00%)	0.013
*DSEL*	3/7 (42.86%)	0/19 (0.00%)	0.013
*NSD3*	3/7 (42.86%)	0/19 (0.00%)	0.013
*TSPYL2*	3/7 (42.86%)	0/19 (0.00%)	0.013
*UVSSA*	3/7 (42.86%)	0/19 (0.00%)	0.013
*HLA-DQB2*	6/7 (85.71%)	6/19 (31.58%)	0.026
*COL4A4*	3/7 (42.86%)	1/19 (0.05%)	0.047
*IGSF1*	3/7 (42.86%)	1/19 (0.05%)	0.047
*MLYCD*	3/7 (42.86%)	1/19 (0.05%)	0.047
*PRKDC*	3/7 (42.86%)	1/19 (0.05%)	0.047
*SVIL*	3/7 (42.86%)	1/19 (0.05%)	0.047
*TARBP1*	3/7 (42.86%)	1/19 (0.05%)	0.047
*TG*	3/7 (42.86%)	1/19 (0.05%)	0.047

* Comparing patients with advanced-stage WT (COG stage III–V) who are resistant to chemotherapy with those who are responsive.

## Data Availability

The data presented in this study are available on request from the corresponding author due to ethical concerns regarding patient privacy.

## Supplementary Materials

The following supporting information can be downloaded via this link: https://www.mdpi.com/article/10.3390/cancers16081567/s1, Supplementary Figure S1: The distribution of driver mutations in chemotherapy-resistant and chemotherapy-responsive WT patients; Supplementary Table S1: The mutation rate in the genes associated with clinicopathological features; Supplementary Table S2: The potential targeted drugs for the driver mutations in chemotherapy-resistant WT; Supplementary Data: The dosage of chemotherapy administered to the patients in the resistant group; Supplementary Material: Collaborators with/Membership of the TPCA Consortium.
