# Supplementary material for: Discovery of Novel Potential Prognostic Markers and Targeted Therapy to Overcome Chemotherapy Resistance in an Advanced-Stage Wilms Tumor
Source: Cancers (Basel). 2024 Apr 19;16(8):1567. doi: 10.3390/cancers16081567 (PMC11049388; doi:10.3390/cancers16081567)
Supplement: Supplementary file 1 [file cancers-16-01567-s001.zip › Supplementary data.pdf]

| Sample ID | COG stage | Histology | Chemotherapy regimen |
|---|---|---|---|
| A055 | III | Favorable | ThaiPOG-WT-1302 |
| A159 | III | Favorable | |
| A171 | III | Favorable | |
| A342 | III | Favorable | |
| A265 | IV | Unfavorable | ThaiPOG-WT-1303 |
| A458 | IV | Unfavorable | |
| A203 | V | Unfavorable | ThaiPOG-WT-1305 |

| **Drug** | **Dosage** | | |
|---|---|---|---|
| | **Age < 12 months** | **Age ≥ 12 months** | **BW ≥ 30 kg** |
| **Regimen : ThaiPOG-WT-1302** | | | |
| AMD* = Actinomycin-D X 1 day<br>Begin within 5 days post surgery | 0.023 mg/kg IV | 0.045 mg/kg IV | 1.35 mg/m$^2$ IV<br>(Maximum dose 2.3 mg) |
| VCR1 = Vincristine X 1 day<br>Begin within 7 days post surgery | 0.025 mg/kg IV | 0.05 mg/kg IV | 1.5 mg/m$^2$ IV<br>(Maximum dose 2 mg) |
| VCR2 = Vincrintine X 1 day | 0.034 mg/kg IV | 0.067 mg/kg IV | 2 mg/m$^2$ IV<br>(Maximum dose 2 mg) |
| DOX1* = Doxorubicin X 1 day | 0.75 mg/kg IV | 1.5 mg/kg IV | 45 mg/m$^2$ IV |
| DOX2 = Doxorubicin X 1 day | 0.5 mg/kg IV | 1 mg/kg IV | 30 mg/m$^2$ IV |
| * Actinomycin-D and Doxorubicin doses should be decreased 50% during radiation therapy. | | | |
| **Regimen : ThaiPOG-WT-1303** | | | |
| DOX* = Doxorubicin X 1 day<br>Begin within 5 days post surgery | 0.75 mg/kg IV | 1.5 mg/kg IV | 45 mg/m$^2$ IV |
| VCR1 = Vincristine X 1 day<br>Begin within 7 days post surgery | 0.025 mg/kg IV | 0.05 mg/kg IV | 1.5 mg/m$^2$ IV |
| VCR2 = Vincristine X 1 day | 0.034 mg/kg IV | 0.067 mg/kg IV | 2 mg/m$^2$ IV<br>(Maximum dose 2 mg) |
| CTX5** = Cyclophophamide X 5 days | 7.35 mg/kg/day | 14.7 mg/kg/day | 440 mg/m$^2$/day |
| CTX3** = Cyclophophamide X 3 days | | | |
| ETOP = Etoposide X 5 days | 1.65 mg/kg/day | 3.3 mg/kg/day | 100 mg/m$^2$/day |
| * Doxorubicin doses should be decreased 50% during radiation therapy.<br>** G-CSF 5 mcg/kg/day starting 24 hours after CTX3/CTX5 until ANC recovery. | | | |

| Drug | Dosage | | |
|---|---|---|---|
| | **Age < 12 months** | **Age ≥ 12 months** | **BW ≥ 30 kg** |
| **Regimen : ThaiPOG-WT-1305** | | | |
| DOX* = Doxorubicin IV over 15-120 min on day 1 | 1.5 mg/kg/day | 45 mg/$m^2$/day | 45 mg/$m^2$/day |
| VCR = Vincristine IV on day 1 (Maximum dose 2 mg) | 0.025 mg/kg IV | 0.05 mg/kg IV | 1.5 mg/$m^2$ IV |
| CTX1 = Cyclophophamide IV over 1 hr on Day 1-4 | 14.7 mg/kg/day | 440 mg/$m^2$/day | 440 mg/$m^2$/day |
| CTX2 = Cyclophophamide IV over 1 hr on Day 1 | 40 mg/kg/day | 1,200 mg/$m^2$/day | 1,200 mg/$m^2$/day |
| Carbo = Carboplatin IV over 1 hr on Day 1 Dose based on GFR | See carboplatin dosage adjustment below | | |
| ETOP = Etoposide IV over 1 hr on day 1-4 | 3.3 mg/kg/day | 100 mg/$m^2$/day | 100 mg/$m^2$/day |
| * Doxorubicin doses should be decreased 50% during radiation therapy. | | | |

| **Carboplatin IV over 1 hr** | |
|---|---|
| **GFR ( mL/min/1.73$m^2$)** | **Dose** |
| >150 | 560 mg/$m^2$ (18.7 mg/kg if <1 yr) |
| 100-150 | 500 mg/$m^2$ (16.6 mg/kg if <1 yr) |
| 75-99 | 370 mg/$m^2$ (12.3 mg/kg if <1 yr) |
| 50-74 | 290 mg/$m^2$ (9.7 mg/kg if <1 yr) |
| 30-49 | 200 mg/$m^2$ (6.7 mg/kg if <1 yr) |
| <30 | Hold Carboplatin |
