# Supplementary material for: Discovery of Novel Potential Prognostic Markers and Targeted Therapy to Overcome Chemotherapy Resistance in an Advanced-Stage Wilms Tumor
Source: Cancers (Basel). 2024 Apr 19;16(8):1567. doi: 10.3390/cancers16081567 (PMC11049388; doi:10.3390/cancers16081567)
Supplement: Supplementary file 1 [file cancers-16-01567-s001.zip › Supplementary Material.pdf]

Supplementary Material: Collaborators/Membership of the Thai Pediatric Cancer Atlas (TPCA) Consortium

Co-chairs: Suradej Hongeng[1], Usanarat Anurathapan[1], Somchai Chutipongtanate[2], Surasak Sangkhathat[3,4], Dumnoensun Pruksakorn[5]

Tissue and clinical data source sites: Mahidol University Usanarat Anurathapan[1], Natini Jinawath[6], Praewa Suthapot[1], Nilubon Singhto[6]; **Prince of Songkla University** Surasak Sangkhathat[3,4], Pongsakorn Choochuen[3,4], Wanwisa Maneechay[7], Yanisa klaewtanong[4,3], Wararak Kaewrattana[4,3]; **Chiang Mai University** Dumnoensun Pruksakorn[5], Pimpisa Teeyakasem[8]

Cancer multi-omics data centers: Mahidol University Wararat Chiangjong[9], Praewa Suthapot[1], Jirawan Weeraphan[1], Nutkridta Pongsakul[9]; **Prince of Songkla University** Pongsakorn Choochuen[3,4], Natakorn Nokchan[3,4], Natthapon Khongcharoen[3,4], Yanisa klaewtanong[4,3], Wararak Kaewrattana[4,3]; **Chiang Mai University** Parunya Chaiyawat[5], Nutnicha Sirikaew[10], Viraporn Thepbundit[10], Petlada Yongpitakwattana[5]

Data analysis subgroup: Mahidol University Wararat Chiangjong[9], Praewa Suthapot[1], Jirawan Weeraphan[1], Nutkridta Pongsakul[9]; **Prince of Songkla University** Pongsakorn Choochuen[3,4], Natakorn Nokchan[3,4], Natthapon Khongcharoen[3,4]; **Chiang Mai University** Nutnicha Sirikaew[10], Jeerawan Klangjorhor[5,11], Sutpirat Moonmuang[5,11], Tanyaluck Kampoun[5], Viraporn Thepbundit[10], Sasimol Udomruk[5], Santhasiri Orrapin[5], Warunyoo Phannasorn[5], Thanapak Jaimalai[5]

[1]Division of Hematology and Oncology, Department of Pediatrics, Faculty of Medicine Ramathibodi Hospital, Mahidol University, Bangkok 10400, Thailand. [2]Division of Epidemiology, Department of Environmental and Public Health Sciences, University of Cincinnati College of Medicine, Cincinnati,

OH 45267, USA. [3]Department of Biomedical Sciences and Biomedical Engineering, Faculty of Medicine, Prince of Songkla University, Songkhla 90110, Thailand. [4]Translational Medicine Research Center, Faculty of Medicine, Prince of Songkla University, Songkhla 90110, Thailand. [5]Center of Multidisciplinary Technology for Advanced Medicine (CMUTEAM), Faculty of Medicine, Chiang Mai University, Chiang Mai 50200, Thailand. [6]Ramathibodi Comprehensive Cancer Center, Faculty of Medicine Ramathibodi Hospital, Mahidol University, Bangkok 10400, Thailand. [7]Innovation Center, Faculty of Medicine, Prince of Songkla University, Songkhla 90110, Thailand. [8]Research Administration Section, Faculty of Medicine, Chiang Mai University, Chiang Mai 50200, Thailand. [9]Pediatric Translational Research Unit, Department of Pediatrics, Faculty of Medicine Ramathibodi Hospital, Mahidol University, Bangkok 10400, Thailand. [10]Department of Biochemistry, Faculty of Medicine, Chiang Mai University, Chiang Mai 50200, Thailand. [11]Office of research administration, Chiang Mai University, Chiang Mai 50200, Thailand.
