# Supplementary material for: Discovery of Novel Potential Prognostic Markers and Targeted Therapy to Overcome Chemotherapy Resistance in an Advanced-Stage Wilms Tumor
Source: Cancers (Basel). 2024 Apr 19;16(8):1567. doi: 10.3390/cancers16081567 (PMC11049388; doi:10.3390/cancers16081567)
Supplement: Supplementary file 1 [file cancers-16-01567-s001.zip › Supplementary Table S1.pdf]

Supplementary Table S1 Genes associated with clinicopathological features			
Genes	Histology		p-value
	Unfavorable (N:7)	Favorable (N:36)	
MMP17	3/7 (42.86%)	0/36 (0.00%)	0.003
OR6C70	3/7 (42.86%)	0/36 (0.00%)	0.003
MPDZ	4/7 (57.14%)	2/36 (5.56%)	0.004
FLG2	3/7 (42.86%)	1/36 (2.78%)	0.010
KIF13A	3/7 (42.86%)	1/36 (2.78%)	0.010
KRT6B	3/7 (42.86%)	1/36 (2.78%)	0.010
MEGF8	3/7 (42.86%)	1/36 (2.78%)	0.010
RFC1	3/7 (42.86%)	1/36 (2.78%)	0.010
APOB	4/7 (57.14%)	4/36 (11.11%)	0.015
CMYA5	4/7 (57.14%)	4/36 (11.11%)	0.015
ATG2A	3/7 (42.86%)	2/36 (5.56%)	0.024
CNTNAP3B	3/7 (42.86%)	2/36 (5.56%)	0.024
EXD3	3/7 (42.86%)	2/36 (5.56%)	0.024
NBPF15	3/7 (42.86%)	2/36 (5.56%)	0.024
PKD1	3/7 (42.86%)	2/36 (5.56%)	0.024
SAMD11	3/7 (42.86%)	2/36 (5.56%)	0.024
WDR87	3/7 (42.86%)	2/36 (5.56%)	0.024
GOLGA6L9	5/7 (71.43%)	10/36 (27.78%)	0.040
AKAP11	3/7 (42.86%)	3/36 (8.33%)	0.045
CFAP54	3/7 (42.86%)	3/36 (8.33%)	0.045
EML6	3/7 (42.86%)	3/36 (8.33%)	0.045
PTPN13	3/7 (42.86%)	3/36 (8.33%)	0.045
TEX15	3/7 (42.86%)	3/36 (8.33%)	0.045
Genes	Syndromic feature		p-value
	Yes (N:10)	No (N:33)	
PRSS2	7/10 (70.00%)	6/33 (18.18%)	0.004
PRSS1	7/10 (70.00%)	7/33 (21.21%)	0.007
ATG14	4/10 (40.00%)	1/33 (3.03%)	0.007
CEP170B	4/10 (40.00%)	1/33 (3.03%)	0.007
AKR7A3	3/10 (30.00%)	0/33 (0.00%)	0.010
ARID1A	3/10 (30.00%)	0/33 (0.00%)	0.010
C20orf144	3/10 (30.00%)	0/33 (0.00%)	0.010

DENND1C	3/10 (30.00%)	0/33 (0.00%)	0.010
FAM107A	3/10 (30.00%)	0/33 (0.00%)	0.010
GAA	3/10 (30.00%)	0/33 (0.00%)	0.010
METTL14	3/10 (30.00%)	0/33 (0.00%)	0.010
PAMR1	3/10 (30.00%)	0/33 (0.00%)	0.010
TNIK	3/10 (30.00%)	0/33 (0.00%)	0.010
ACSM2A	4/10 (40.00%)	2/33 (6.06%)	0.020
AKNA	4/10 (40.00%)	2/33 (6.06%)	0.020
NBPF4	4/10 (40.00%)	2/33 (6.06%)	0.020
ABCC12	3/10 (30.00%)	1/33 (3.03%)	0.034
ATP10B	3/10 (30.00%)	1/33 (3.03%)	0.034
BRD8	3/10 (30.00%)	1/33 (3.03%)	0.034
CEP295	3/10 (30.00%)	1/33 (3.03%)	0.034
CES5A	3/10 (30.00%)	1/33 (3.03%)	0.034
CIT	3/10 (30.00%)	1/33 (3.03%)	0.034
CRYBG3	3/10 (30.00%)	1/33 (3.03%)	0.034
DAG1	3/10 (30.00%)	1/33 (3.03%)	0.034
DAZAP2	3/10 (30.00%)	1/33 (3.03%)	0.034
FAM214B	3/10 (30.00%)	1/33 (3.03%)	0.034
GAK	3/10 (30.00%)	1/33 (3.03%)	0.034
IFNL1	3/10 (30.00%)	1/33 (3.03%)	0.034
MYH15	3/10 (30.00%)	1/33 (3.03%)	0.034
MYH4	3/10 (30.00%)	1/33 (3.03%)	0.034
MYH7B	3/10 (30.00%)	1/33 (3.03%)	0.034
NUP188	3/10 (30.00%)	1/33 (3.03%)	0.034
SUN1	3/10 (30.00%)	1/33 (3.03%)	0.034
SVEP1	3/10 (30.00%)	1/33 (3.03%)	0.034
SYN3	3/10 (30.00%)	1/33 (3.03%)	0.034
KRTAP5-7	8/10 (80.00%)	13/33 (39.39%)	0.034
CHD8	4/10 (40.00%)	3/33 (9.09%)	0.040
PCDHB14	4/10 (40.00%)	3/33 (9.09%)	0.040
Genes	Laterality		p-value
	Bilateral (N:5)	Unilateral (N:38)	
AKR7A3	3/5 (60.00%)	0/38 (0.00%)	0.001
ARID1A	3/5 (60.00%)	0/38 (0.00%)	0.001

METTL14	3/5 (60.00%)	0/38 (0.00%)	0.001
BRD8	3/5 (60.00%)	1/38 (2.63%)	0.003
ZNF683	3/5 (60.00%)	2/38 (5.26%)	0.008
PRSS2	4/5 (80.00%)	9/38 (23.68%)	0.023
BTBD7	3/5 (60.00%)	4/38 (10.53%)	0.024
CHD8	3/5 (60.00%)	4/38 (10.53%)	0.024
PRSS1	4/5 (80.00%)	10/38 (26.32%)	0.032
GLOD4	3/5 (60.00%)	5/38 (13.16%)	0.037
