# Supplementary material for: Discovery of Novel Potential Prognostic Markers and Targeted Therapy to Overcome Chemotherapy Resistance in an Advanced-Stage Wilms Tumor
Source: Cancers (Basel). 2024 Apr 19;16(8):1567. doi: 10.3390/cancers16081567 (PMC11049388; doi:10.3390/cancers16081567)
Supplement: Supplementary file 1 [file cancers-16-01567-s001.zip › Supplementary Table S2.pdf]

Supplementary Table S2 The potential targeted drugs for the driver mutations in chemotherapy-resistant WT

Gene	Amino acid change	Potential chemotherapy	Disease*	Response	Level of evidence#
KIT	p.L862M	KIT inhibitor (Imatinib)<br>KIT inhibitor + Pan-TK inhibitor (Sunitinib + Sorafenib)<br>KIT inhibitor + PI3K inhibitor (Imatinib + Pictilisib)	Melanoma<br>Thymic cancer<br>GIST	Responsive	B<br>B<br>D
PALB2	p.M723X	PARP inhibitor (Olaparib)<br>PARP inhibitor<br>Antibiotic/antineoplastic agent (Mytomycin C)<br>Platinum-based Agent	Prostate cancer<br>Pancreatic cancer<br>Pancreatic cancer<br>Pancreatic cancer	Responsive	A<br>D<br>C<br>C
LRP1B	p.S1148P (A159),<br>p.W3333L (A203)	Antibiotic/antineoplastic agent (Liposomal Doxorubicin)	Ovarian cancer	Resistant	C
SMAD4	p.N369S	EGFR mAb inhibitor (Panitumumab + Cetuximab)	CRC	Resistant	B
CDH1	p.D433G	AR inhibitor (Bicalutamide)	Breast cancer	Responsive	D
BRCA1	p.Q262H	PARP inhibitor (Talazoparib, Olaparib)<br>PARP inhibitor (Niraparib, Olaparib, Rucaparib)<br>PARP inhibitor (Olaparib, Rucaparib)<br>Combined PARP inhibitor (Talazoparib + Olaparib)<br>PARP inhibitor + VEGF mAb inhibitor (Olaparib + Bevacizumab)<br>PARP inhibitor + VEGF inhibitor (Cediranib + Olaparib)<br>Combine platinum-based agent (Carboplatin + Cisplatin)<br>Platinum-based agent (Carboplatin, Cisplatin)<br>PARP inhibitor + Platinum-based agent (Veliparib + Cisplatin)<br>WEE1 inhibitor	Breast cancer<br>Ovarian cancer<br>Prostate cancer<br>Breast cancer<br>Ovarian cancer<br>Ovarian cancer<br>Breast cancer<br>Ovarian cancer<br>Breast cancer<br>Any cancer type	Responsive	A, B<br>A, B, B<br>A, A<br>B<br>A<br>B<br>B<br>B<br>C<br>C
CTNNB1	p.S45F	Tankyrase inhibitors	CRC	Resistant	D
DNMT3A	p.V687F	Antibiotic/antineoplastic agent (Daunorubicin)<br>Nucleoside analog (Decitabine)	AML<br>AML	Responsive	A<br>B
		Combined PD1 Ab inhibitors (Pembrolizumab + Nivolumab + Atezolizumab)	Any cancer types	Resistant	B
NF1	p.P1421Q	MTOR inhibitor (Everolimus)*<br>MEK inhibitors (Selumetinib)<br>MEK inhibitors (Trametinib)<br>VEGFR mAb inhibitor (Bevacizumab)	Neurofibroma<br>PNF<br>Glioma<br>Glioma	Responsive	B<br>B<br>C<br>C

		MTOR inhibitor + VEGFR inhibitor (Everolimus + Pazopanib)	HCC		C
		MTOR inhibitor + EGFR inhibitor 1st gen (Sirolimus + Erlotinib)	Glioma		C
		KIT inhibitor (Imatinib)	MPNST		C
		PD1 Ab inhibitors	Melanoma		C
		Tubulin inhibitors (Vinblastine)	Glioma		C
		Tubulin inhibitors + BCR-ABL inhibitor 2nd gen (Vinblastine + Nilotinib)	Glioma		C
		AURK inhibitors	MPNST		D
		BRD4 inhibitors	MPNST		D
		MEK inhibitors (Cobimetinib + Trametinib)	Any cancer types		D
		KIT inhibitor + MTOR inhibitors	MPNST		D
		MTOR inhibitor + HSP90 inhibitors	MPNST		D
		BCR-ABL inhibitor 2nd gen (Nilotinib)	PNF, MPNST		D
		Pan-TK inhibitor (PLX3397)	PNF		D
		MEK inhibitors + Pan-RAF inhibitor	Melanoma		D
		MTOR inhibitor + MEK inhibitors (Rapamycin + Sirolimus + 391210-10-9)	Melanoma		D
		MTOR inhibitor + Pan-TK inhibitor (Sorafenib + Sirolimus)	MPNST		D
		Hormonal agent (Tamoxifen)	MPNST		D
		BRAF inhibitor (Vemurafenib)	Melanoma	Resistant	C
		BCR-ABL inhibitor (Dasatinib)	Lung cancer		D
		EGFR inhibitor (Erlotinib)	Lung cancer		D
		Retinoic Acids	Neuroblastoma		D
PBRM1	p.G989C	EZH2 inhibitors	Any cancer types	Responsive	D
ERCC6	p.M867V	Platinum-based agent (Cisplatin)	Ovarian cancer	Responsive	D
FBXW7	p.R505G (A265), p.Q492QX (A265)	Steroids	ALL	Responsive	B
		MTOR inhibitor	Any cancer types		B
		EGFR mAb inhibitor (Panitumumab + Cetuximab)	CRC	Resistant	B
		Tubulin inhibitors	Any cancer types		D
SMARCB1	p.R53*	EZH2 inhibitors	MRT	Responsive	C
		HDAC inhibitors	MRT		D
TP53	p.R273C	CD52 mAb antibody (Alemtuzumab)	CLL	Responsive	B
		Antibiotic/antineoplastic agent (Doxorubicin)	Bladder cancer		B
		ATR inhibitor (AZD6738)	BCL		C

		Nucleoside analog (Decitabine)	AML, MPS		C
		WEE1 inhibitors	Ovarian cancer		C
		2-Aminoethyl Dihydrogen Phosphate	Stomach cancer		C
		Antibiotic/antineoplastic agent (Mytomycin C)	Bladder cancer		D
		Amylin analogue (Pramlintide)	Thymic cancer		D
		MEK inhibitors + Antibiotic/antineoplastic agent (Selumetinib + Docetaxel)	NSLC		D
		MDM2 inhibitors	Liposarcoma	Resistant	C
		CDK4/CDK6 inhibitor (Abemaciclib)	Breast cancer		C
		Platinum-based agent (Cisplatin)	GCT		C
		Hormonal agent (Tamoxifen)	Breast cancer		C
		Antibiotic/antineoplastic agent (Docetaxel)	NSLC		D

*GIST = Gastrointestinal stromal tumors, HCC = Hepatocellular carcinoma, MPNST = Malignant peripheral nerve sheat tumor, PNF = Plexiform neurofibroma, CRC = Colorectal cancer, MRT = Malignant rhabdoid tumor, CLL = Chronic lymphocytic leukemia, ALL = Acute lymphoblastic leukemia, BCL = B cell lymphoma, AML = Acute myeloid leukemia, MPS = Myelodisplasic proliferative syndrome, NSLC = Non-small cell lung cancer, GCT = Germ cell tumor

#Level of evidence are obtained from the VICC integrated knowledge base, and are classified as follow : Level A - corresponds to biomarkers used in professional guidelines of FDA approved drugs, Level B - groups biomarkers observed in clinical trial, Level C -corresponds to biomarkers identified from small group studies or case studies, and Level D - biomarkers have been identified in pre-clinical studies.
