# Supplementary material for: Discovery of Novel Potential Prognostic Markers and Targeted Therapy to Overcome Chemotherapy Resistance in an Advanced-Stage Wilms Tumor
Source: Cancers (Basel). 2024 Apr 19;16(8):1567. doi: 10.3390/cancers16081567 (PMC11049388; doi:10.3390/cancers16081567)
Supplement: Supplementary file 1 [file Supplementary Figure S1.pdf › Supplementary Figure S1.pdf]

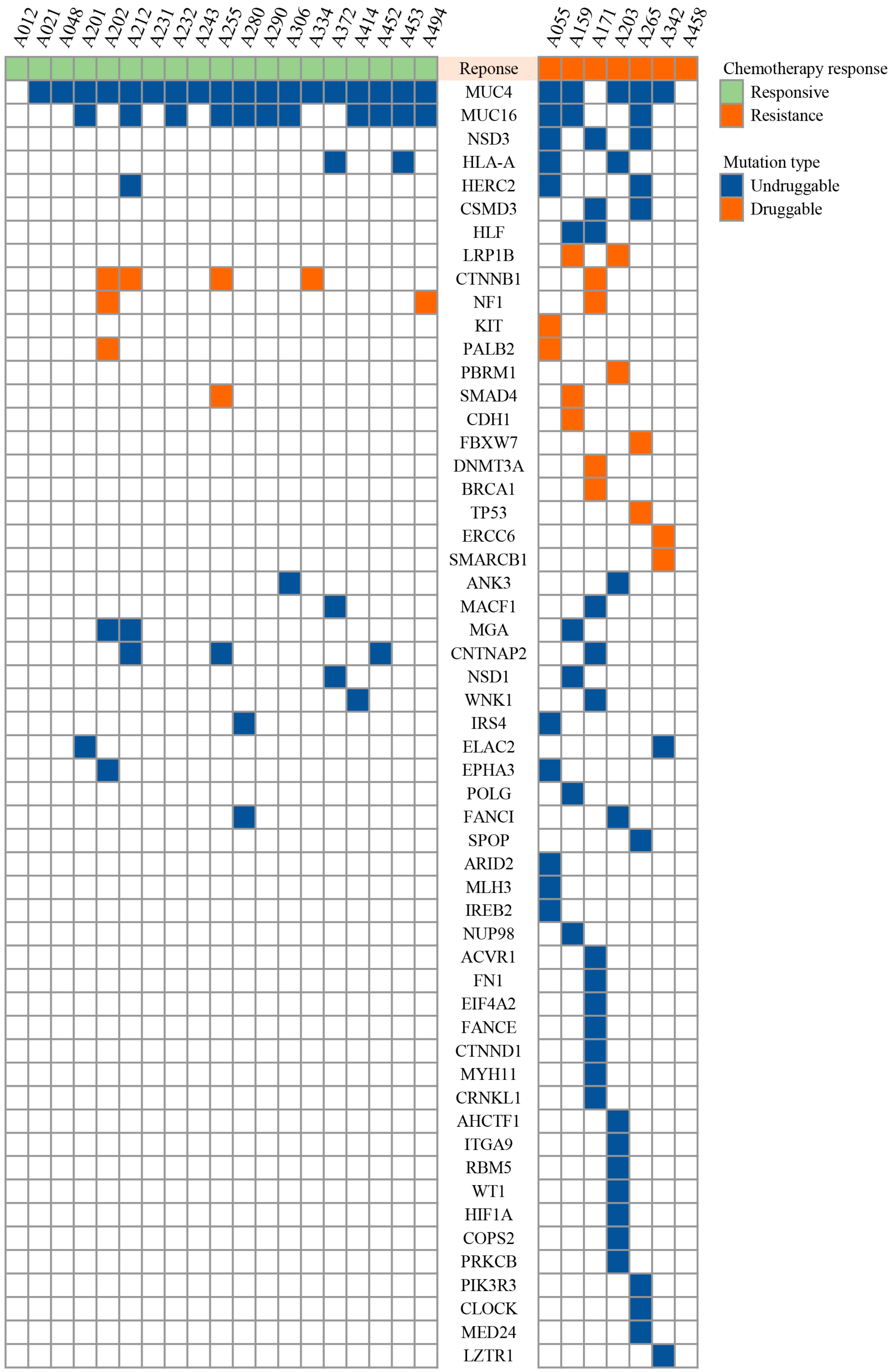

Figure S1. The distribution of driver mutations in chemotherapy-resistant and chemotherapy-responsive WT patients.
